# Supplementary material for: Content-rich biological network constructed by mining PubMed abstracts
Source: BMC Bioinformatics. 2004 Oct 8;5:147. doi: 10.1186/1471-2105-5-147 (PMC528731; doi:10.1186/1471-2105-5-147)
Supplement: Additional File 2 — The original results of the above study (non-essential files are deleted to keep the file size under the limit set by BMC bioinformatics). [file 1471-2105-5-147-S2.bz2 › chilibotAdditionalFile2/dip20/411ID7673243E10074/html/BMP4_BMPR1A.html]

 


 **BMP4** and **BMPR1A** 
  
Found 15 abstracts in PubMed, retrieved 15.  
 

 What does Google say? 
 PDF only 
| .edu only 

---

**Interactive relationship** (e.g. stimulation, inhibition, etc)

**Neutral relationship**- ... BMP 4  [ **BMP4** ]  bound to ALK 3  [ **BMPR1A** ]  and ALK 6 efficiently.  Ref: 8006002 J Biol Chem, 1994
- BMP 4  [ **BMP4** ]  bound to ALK 3  [ **BMPR1A** ]  in MC3T3 E1 osteoblasts and human foreskin fibroblasts.  Ref: 8006002 J Biol Chem, 1994
- Immunoprecipitation of the ALK 3  [ **BMPR1A** ]  protein cross linked to 125I BMP 4  [ **BMP4** ]  revealed two major receptor complexes of approximately 90 kDa and 170 kDa in size.  Ref: 8707881 J Cell Physiol, 1996
- These results suggest that ALK 3  [ **BMPR1A** ]  and ALK 6 are type I receptors for OP 1 and BMP 4  [ **BMP4** ] .  Ref: 8006002 J Biol Chem, 1994
- BMP 4  [ **BMP4** ]  and GDF 5 are known to bind to activin receptor like kinase 3  [ **BMPR1A** ]  ALK 3  [ **BMPR1A** ]  and or ALK 6 also termed BMP type IA and type IB receptors, respectively.  Ref: 11282024 J Cell Sci, 2001
- The BMP 4  [ **BMP4** ]  transcripts were expressed in all stages of differentiation, as were transcripts for BMP type IA receptor ALK 3  [ **BMPR1A** ]  and BMP type II receptor.  Ref: 10984103 Cell Struct Funct, 2000
- Bmpr, also known as ALK 3  [ **BMPR1A** ]  and Brk 1, encodes a type I transforming growth factor beta TGF beta family receptor for BMP 2 and BMP 4  [ **BMP4** ] .  Ref: 8543149 Genes Dev, 1995
- ALK 5 is a TGF beta type I receptor, ALK 2 and ALK 4 are activin type I receptors, and ALK 3  [ **BMPR1A** ]  and ALK 6 are type I receptors for osteogenic protein 1 OP 1 bone morphogenetic protein 7 BMP 7 and BMP 4  [ **BMP4** ] .  Ref: 7750489 Endocrinology, 1995

**Non-interactive relationship** (e.g. studied together, co-existance, homology, etc.)

- Developmental expression of  **BMP4**  ALK3  [ **BMPR1A** ]  SMAD5 signaling pathway in the mouse testis a potential role of  **BMP4**  in spermatogonia differentiation.  Ref: 12857787 J Cell Sci, 2003
- These results suggest that the differentiation of pulp cells into preodontoblasts may be regulated by changes in the temporally coordinated expression pattern of TGF beta superfamily members and their receptors, including up regulation of transcription of TGF beta 1, BMP 4  [ **BMP4** ] , BMP 7, ALK 2, ALK 3  [ **BMPR1A** ] , and ALK 5.  Ref: 9296267 Arch Oral Biol, 1997
- To explore the molecular cascade of odontoblast differentiation, Northern blot analyses and reverse transcriptase polymerase chain reaction were here used to investigate the expression patterns of the genes for TGF beta superfamily members TGF beta 1, namely bone morphogenetic protein BMP 4  [ **BMP4** ] , BMP 7, activin beta A and activin beta B, and their type I and type II receptors, namely activin receptor like kinase ALK 2 ActR I, ALK 3  [ **BMPR1A** ]  BMPR IA, ALK 4 ActR IB, ALK 5 T beta R I, BMPR II and T beta R II, during differentiation of pulp cells into preodontoblasts in bovine adult pulp cell culture.  Ref: 9296267 Arch Oral Biol, 1997
- Similar to  **BMP4**  and ALK3  [ **BMPR1A** ]  mutants, the mutant embryos display severe defects in primitive streak and mesoderm formation.  Ref: 9550720 Development, 1998
- In the present study, we examine the effect of  **BMP4**  on the development of metanephric and periureteral mesenchymal cells, which express the BMP type I receptor gene,  **Bmpr1a**  Alk3  [ **BMPR1A** ] .  Ref: 12631064 Kidney Int, 2003
